# Discordance in Addressing Opioid Crisis in Rural Communities: Patient and Provider Perspectives

**DOI:** 10.3390/pharmacy10040091

**Published:** 2022-07-27

**Authors:** Bonyan Qudah, Martha A. Maurer, David A. Mott, Michelle A. Chui

**Affiliations:** 1 Department of Social and Administrative Sciences, School of Pharmacy, University of Wisconsin, Madison, 777 Highland Ave., Madison, WI 53705, USA; david.mott@wisc.edu (D.A.M.); michelle.chui@wisc.edu (M.A.C.); 2 Sonderegger Research Center, School of Pharmacy, University of Wisconsin-Madison School of Pharmacy, 777 Highland Ave, Madison, WI 53705, USA; mamaurer@wisc.edu

**Keywords:** opioid use disorder, chronic pain, rural, medications for opioid use disorder, stigma

## Abstract

Providing patient-centered care to manage chronic pain and opioid use disorder (OUD) is associated with improved health outcomes. However, adopting a holistic approach to providing care is often challenging in rural communities. This study aims to identify and contrast challenges to providing patient-centered care from the perspective of patients and providers. A participatory design approach was adopted to elicit the perceptions of providers and patients with lived experiences of chronic pain and/or OUD in Jefferson County, Wisconsin. Two focus groups were conducted with each stakeholder group to identify problems that participants face with respect to chronic pain management and OUD and possible solutions. Four interviews were conducted with providers experienced in chronic pain management. Analysis of focus group sessions and interviews show consensus among patients and providers that lack of behavioral health and recovery resources create barriers to effectively manage OUD and chronic pain. However, there was discordance among the two groups about other barriers such as patient and provider attitudes, tapering approach, and access to medications for OUD. This tension among patients and providers can influence patients’ retention in therapy. More efforts are needed to mitigate stigma among providers in rural communities and support psychosocial needs of patients.

## 1. Background

Chronic pain and opioid use disorder are intertwined public health problems that continue to grow in the United States despite advances in the field of medicine [1]. More than 90,000 Americans died of opioid overdose in 2020, representing a 30% increase compared to 2019. The outbreak of COVID-19 in 2020 worsened the opioid crisis, as many people lost their jobs and grappled with stress and isolation [2]. The economic cost of opioid use disorder (OUD) and fatal opioid overdose in the United States totaled $1 trillion in 2017. In Wisconsin, the economic burden was estimated at $19 billion [3]. Chronic pain affects about 20% of the US population^4^. Inadequate chronic pain treatment was associated with disability, reduced employment, worsened mental health, and lower quality of life [4,5].

The opioid epidemic has disproportionate impacts on some rural communities. Opioid-related mortality quadrupled in rural areas between 1999 and 2015 [6]. Although both rural and urban areas in Wisconsin are affected by the opioid epidemic, inadequate availability of medications for opioid use disorder (MOUD) in rural counties can have dire consequences on preventing and treating the harms of the opioid crisis [7,8]. Factors that exacerbated the opioid crisis in rural communities include increased access to opioid prescriptions, lower employment opportunities, and economic insecurity [9]. Moreover, patients experience higher levels of stigma in rural communities due to difficulty maintaining anonymity and higher likelihood of having friends or relatives who work as service providers [9]. The level of physicians’ bias towards patients with OUD in one rural area exceeded the bias reported in literature towards injecting drug users, patients with Hepatitis C, and injecting drug users infected with HIV [10]. It is concerning that providers are showing greater bias toward patients in communities that already have limited healthcare facilities and recovery resources for OUD [11]. Providers’ stigma and mutual distrust create tension between patients and providers and ultimately may reduce the likelihood that patients initially seek and continue using opioid replacement treatments. Patients with chronic pain have also reported that they face stigma from their healthcare providers [12]. Providers were less inclined to help and had lower sympathy towards with patients with chronic pain due to their fear of patients’ diversion and misuse in the face of the opioid epidemic [13].

Despite the interest shown by patients with chronic pain and/or OUD to be involved in the management processes and receiving individualized and holistic care, chronic pain management and OUD have not consistently been addressed using a patient-centered approach [14,15]. Previous studies showed that health system-centric outcome measures for substance use disorder overlook many of the concerns that are relevant to patients and their journey with addiction [16]. Providing patient-centered care was correlated with higher trust in providers, improved health outcomes and reduced utilization of health care services [17,18].

Identifying challenges to effectively managing patients with chronic pain and/or OUD from both the patient and provider perspectives is important to understand whether patients and providers have similar or antagonistic views of these therapeutic situations. In one study, tension was noted between patients with chronic pain and providers, and the tension was due to conflicting needs and agendas that are inherent in their roles [14]. It is yet unclear whether this polarity is present in rural communities.

The current study is the result of a partnership between an academic team and a rural health center who collaborated to elicit the perspectives of various stakeholders to inform the planning and development of initiatives to improve opioid stewardship across the care continuum. The collaboration resulted in a series of meetings with various stakeholders in a rural county with the ultimate goal of identifying potential future solutions to address opioid prescribing and improve aspects of the opioid crisis that are most important for the health system and their communities. Although the end goal of the academic–providers partnership was to develop an opioid stewardship intervention, the objective of this sub-study was restricted to exploring patients and providers’ perspectives about challenges to managing the opioid crisis in rural communities, including preventing and treating OUD as well as appropriately managing chronic pain.

## 2. Methods

### 2.1. Overall Study Design

This study used an abbreviated version of a participatory design (PD) approach that involves obtaining input directly from potential users of an intervention when planning the intervention [19,20]. The PD approach referenced by Spinuzzi [19] includes three basic stages: (1) initial exploration of work, (2) discovery process, and (3) prototyping. Participatory design method actively engages end-users in the intervention design through an adaptive cycle of creation and reflection. Evidence suggests that active involvement of patients and providers in PD is well suited for the design of health care interventions that will be implemented in complex work systems that involve diverse groups of patients and providers. The author MC used this approach previously in the design of a health care intervention and found that it led to more in-depth understanding of the reality of problems faced by patients and providers, as well as of considerations regarding the implementation and use of the intervention [21].

### 2.2. Site 

The focus of this study was Jefferson County, a rural county in Southeastern Wisconsin with a population of approximately 85,000 people, that has been severely impacted by the opioid crisis. Fort HealthCare (FHC) represents both the largest healthcare provider and employer in Jefferson County.

### 2.3. Participants

Applying PD in a health system surrounded by, and interconnected with, the broader community presents unique considerations. To capture patient and provider perspectives related to challenges in managing the opioid crisis in Jefferson County, three stakeholder groups were created.

1. Fort HealthCare Providers (FP)—includes FHC providers from multiple specialties who were engaged volunteers on the FHC Opioid Taskforce and invested in addressing the opioid crisis.
2. Patients with lived experience with opioids (PT)—includes patients who were actively managing chronic pain with prescription opioids and patients who were recovering from prescription opioid addiction.
3. Community Stakeholder (CS)—includes members from the community who have strong relationships with FHC leadership and are involved in managing the opioid crisis such as county sheriff, a school district nurse, a community advocate/city council member, independent community pharmacists, public health officials, and patient advocacy group leaders.

Participants from each group were invited to participate in all focus group sessions for their respective group. Only perspectives of the PT and FP stakeholder groups were analyzed and contrasted for this study. We excluded the CS data for this study because this group was heterogenous and comprised of individuals from varied backgrounds and perspectives (e.g., law enforcement) that were not relevant for patient and provider interactions or the clinical management of either chronic pain or OUD. The provider meetings were held at Fort Memorial Hospital. The patient meetings were deliberately held in neutral community venues so that participants would feel comfortable sharing freely about their experiences with FHC. Some meetings were held virtually due to COVID-19.

### 2.4. Participant Recruitment

We used convenience sampling to recruit all participants. Our goal was to recruit 7–8 participants from each of the three stakeholder groups. The study team worked closely with FHC Department of Pharmacy leadership throughout the recruitment process. For the FP group, we recruited members of the current FHC Opioid Taskforce. Recruitment for the PT group involved working with our FHC colleagues to identify individuals with relevant expertise and experience. Additionally, we met with representatives of the Jefferson County Health Department and Human Services to present the project and facilitate recruitment of individuals for the PT group. Study flyers were developed and distributed to collaborators at FHC and through the Jefferson County Drug Free Task Force to facilitate recruitment of individuals for the PT group. Participants interested in joining the study received written informed consent forms that describe the purpose of the study, the procedures, the risks, and how confidentiality would be protected.

### 2.5. Data Collection

The study started in October 2019 and ended in January 2021. We conducted 5 focus groups, two with each stakeholder group and a third focus group with the FP group. Meeting agendas and focus group protocols were developed with the consultation and assistance of the Wisconsin Network for Research Support, a consulting group with expertise in effectively engaging with stakeholders in the community who are highly skilled at providing feedback on recruitment materials and focus group questions [22].

We conducted the focus groups separate from each other because we hypothesized that engagement with and feedback from each stakeholder group would be categorically different. Additionally, we were aware of a potential power differential between the groups, due to the difference in medical expertise that could impact participant comfort levels in providing feedback during sessions. Focus groups were moderated by (MC) and (DM) and notes were taken by (MM). All three researchers have extensive experience in conducting focus groups. All focus groups were audio recorded and later professionally transcribed. Transcripts were deidentified and stored in HIPAA-compliant server.

The purpose of the initial focus group with each stakeholder group was to elicit and understand the problems the stakeholders faced related to the opioid crisis. The goal was to have the participants define the problems on their own terms in a way that was meaningful to them. Various methods were utilized to gather feedback about opioid-related problems such as using sticky notes to record problems anonymously and open discussion. The focus group concluded with discussions about the highest priority problems and potential solutions to address the problems.

Following each of the initial focus groups, the research team reviewed the audio recordings/sticky notes and summarized the problems and solutions identified by each group. The study team then examined the literature to find evidence-based solutions that could be implemented in the community. During the second round of focus groups, the study team presented the proposed solutions and sought feedback on their feasibility, acceptability, and effectiveness from each stakeholder group. At the end of the study, a third focus group was conducted with the FP group to discuss the intervention (s) that were developed with input from the CS and PT groups. Data from the third focus group was not included in the analysis.

### 2.6. Interviews

To supplement the FP focus groups, one member of the team (MM) conducted four individual semi-structured interviews with FHC providers with expertise in treating patients with chronic pain to elicit their input about managing chronic pain and tapering patients on high doses of opioid medications. Interviews were done with a pharmacist, physician assistant, physician, and advanced pain management nurse and were conducted in their workplace at Fort Memorial Hospital and affiliated clinics. All interviews were audio-recorded and transcribed for later analysis.

### 2.7. Thematic Analysis

Data analysis was led by one researcher (BQ), who has experience with conducting thematic analysis, and occurred in two phases. In the first phase, the researcher conducted a first pass by reading the interview and focus group transcripts to familiarize herself with the data and understand the variation in comments made by participants. Then, sections that described challenges to managing chronic pain and treating patients with OUD were highlighted. Open coding was utilized to allow emerging themes to be identified [23]. Categories were developed inductively, which represented coherent accounts of participants perceptions and experiences. Constant comparison and iteration were conducted until final categories and themes were established. Biweekly meetings were conducted with the research team to refine the coding scheme. Through consensus, codes were collapsed into a list of categories and themes representing data from the focus groups and the interviews.

**Figure 1:**
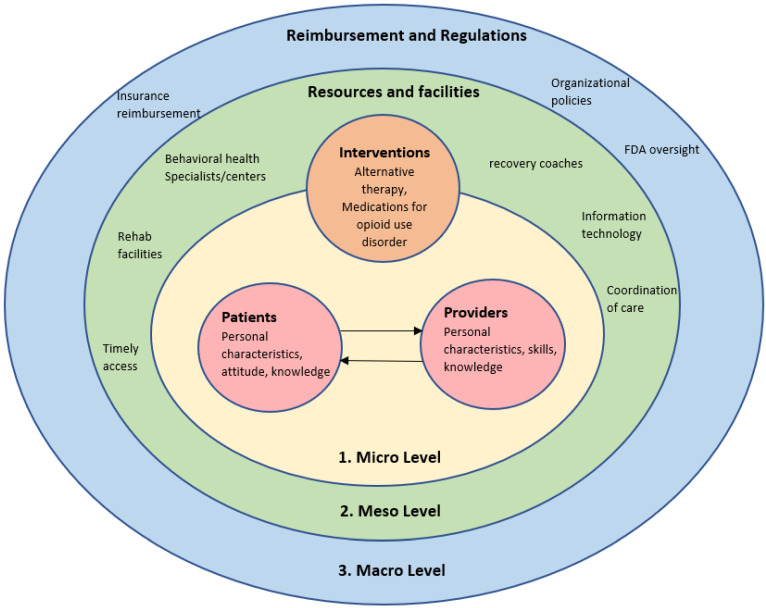
Challenges to providing patient-centered care in the management of OUD.

Main themes reflecting poor communication between patients and providers, poor coordination between different providers, mutual mistrust, patients’ perception of stigma, patients’ resistance to tapering, lack of facilities and recovery resources, and insurance reimbursement issues were initially constructed. As most of the codes reflected the need for holistic care that include patients in decision making, respects their values and needs, and receiving care that is collaborative and accessible, the next step involved revisiting the literature on patient-centered care frameworks. Utilizing a higher-level framework provided a structure for the data and allowed the researcher to make sense of evolving patterns and themes [24]. After discussion and reflection with the research team, a framework by Vennedey et al. [25] that describes barriers and facilitators to patient-centered care was deemed the most suitable synthesizing framework to support a comprehensive view of the challenges that patients and providers face in managing chronic pain and treating patients with OUD. Vennedey et al. [25] classified barriers to patient-centered care based on the three levels of health and social care, which are: microlevel, mesolevel and macrolevel. Barriers at the microlevel of care relate to patient and provider characteristics and responsibilities, available interventions, and wellbeing of providers which, taken together, shape the interaction between a patient and their providers. Barriers at the mesolevel of care are related to processes of care, staffing, and organization infrastructure. Lastly, barriers at the macrolevel of healthcare relate to financing, reimbursement, and laws and regulations. To answer the research question of whether patients and providers share similar or contradicting views of the challenges posed by managing chronic pain and treating OUD, a deductive coding frame based on three levels was constructed [26]. Challenges to providing patient centered care to manage either OUD or chronic pain are summarized in Figure 1. No qualitative analysis software system was used to code the transcripts.

To ensure the rigor of the analysis, several strategies were used. Triangulation was accomplished using data from focus group and interviews for the FP group to ensure the trustworthiness of the findings. Comprehensive description of the study setting, participants, and methods of data collection facilitates the transferability of study findings. Self-reflexivity, which is the process by which researchers evaluate their own biases and motivations and how that might affect their interpretation of the study findings, allowed the researcher involved in the analysis to reflect on whether and how their biases and belief systems would interfere with data interpretation.

## 3. Results

### 3.1. Participants and Setting

Nine providers participated in each of the FP group. Six patients participated in the first PT focus group while five patients attended the second PT focus group. About half (56%) of providers were male and the majority (83%) of individuals in the PT group were female. The FP group was comprised of physicians from multiple specialties (e.g., hospitalist, internal medicine, family medicine, emergency medicine, surgical anesthesia, and pediatrics), an advanced practice surgical anesthesia nurse, pharmacists, and a physical therapist. Individuals in the PT group had varied lived experiences with opioids including use of prescription opioids to manage chronic pain, past experience with OUD, and combined experience of currently taking prescription opioids to manage chronic pain while in recovery from OUD. All study participants from the FP and PT groups were White. All focus groups lasted for 90 min. The four provider interviews lasted approximately 30–45 min.

### 3.2. Summary of Findings

Challenges to managing chronic pain and treating OUD were summarized under each level of health and social care. 

#### 3.2.1. Microlevel: The Interaction between Patient and Providers

- Providers’ knowledge, attitude, and professional skills

Inadequate providers’ knowledge and training in the management of OUD was identified as common problem by both patients and providers. Moreover, both patients and providers articulated that some providers might find difficulty in distinguishing between patients who may be diverting or misusing opioids from those with legitimate needs for treating pain. While patients’ concerns revolved around insufficient providers’ knowledge in MOUD and tapering, providers described limited experience and knowledge in managing complex and relapsing OUD patients.

Although participants in FP were aware of the risks of maintaining patients on opioid medications when they no longer need them, few providers reported that fear of losing patients can sometimes influence their decision to stop or taper opioid medications. Additionally, violent reactions by some patients when treatment discontinuation is discussed by providers lead some providers to diffuse situation by keeping patients on inappropriate opioid regimens.

Participants in the PT group blamed the medical community for the opioid misuse problem and described their frustration regarding the stigma and discrimination that they face during their interactions with health care workers and providers, which was the most prominent theme in PT discussions. Participants experienced stigma from providers in various settings, ranging from primary care clinics to community pharmacies. Although none of the patients who participated in the study were experiencing active OUD, their previous history of OUD and current use of MOUD (i.e., Suboxone) triggered stigmatizing behavior by heath care providers. Patients perceived higher stigma when receiving care from providers in rural settings compared to urban settings. Providers’ stigma manifested as physical distancing from patients and verbally expressing reluctance to prescribe any opioid pain medications, even if patients did not request any opioid pain medications. Patients said that they were provided with information sheets about risks of opioid medication even though they had discontinued opioids a long time ago. Lack of empathy from providers contributed to patients’ demoralization and isolation during their treatment journey for pain and OUD.

b. Patient characteristics and responsibilities

Limited patient knowledge of prescription opioid risks and little benefit achieved by escalating the dose of opioids was cited by providers as a major barrier to effective management of either OUD or chronic pain. Providers attributed poor patient knowledge in OUD and pain management to limited patient education resources and tools (e.g., information sheets or educational videos) and lack of patient awareness about alternative non-opioid therapies. This in turn sets patients’ expectations about getting pain relief from opioid medications only. However, providers shared stories about educating patients about opioid management guidelines and described success in getting patients’ buy-in after providing them with information.

While providers acknowledged patients’ fear of experiencing pain and losing functionality as reasonable to opposing tapering opioid medications, they also alluded to the psychological component of patients’ fear that needs to be addressed through mental health services to facilitate tapering. These challenges were also echoed by patients who expressed their ambivalent feelings towards prescription opioid medications. They recognize the risks of long-term opioid therapy; however they have concerns about the severe withdrawal symptoms and pain flaring that might result from stopping their opioids. Even though patients used the term “addicts” when they were describing themselves and their experiences, they were eager to be seen by the medical community as humans who have mental, physical, and spiritual needs. Patients described their struggle to cope with pain and filling their opioid prescriptions. To avoid being judged and stigmatized, patients demonstrated potentially maladaptive behaviors such as underutilization of prescribed opioids and delaying the refill of opioid prescriptions.

c. Patient-provider communication about treatment goals and tapering

Providers treating patients with high doses of opioid medications for chronic pain described the importance of establishing a relationship and building trust with patients before suggesting opioid tapering. However, they acknowledged the difficulty of establishing a relationship with patients due to lack of effective communication between patients and providers and the delivery of mixed messages about goals of opioid management by different providers. Setting the wrong expectation by telling patients they will experience “zero pain” when trying alternative therapies resulted in setting unrealistic expectations for patients and contributed to medical mistrust. Medical mistrust not only hindered collaboration between patients and their providers, but it was also associated with detrimental health outcomes when patients choose not to disclose their complete medication history to their providers.

Patients perceived mistrust by providers as well. They described being put in situations where they were expected to provide evidence to providers that they were experiencing real pain to justify opioid use. Acute pain was considered more legitimate and received higher attention by providers compared to chronic pain.

All providers described the challenges they face persuading patients to taper their prescription opioid medications. Providers described patients as “hardcore” with “roadblocks”. Patients who were willing to taper doses constituted a very small portion of their patients. Negative patients’ experiences with tapering, taking high doses of opioid medications, long duration of opioid therapy, and having good pain control on current opioid regimen were factors that increased patients’ resistance to tapering and inhibited collaboration with providers. Providers shared their concerns about the negative consequences of tapering discussions such as patients exhibiting violent behavior or canceling appointments, which complicates the management of OUD and chronic pain.

When providers described their communication with patients, there were inconsistencies in their communication style and the extent to which they involve patients in the decision making. Although some providers advocated for shared decision-making and emphasized soliciting patients’ input about the pain management process, other providers called for a provider-centered approach where they impose tapering recommendations on patients. Having a standardized tapering tool for all patients was suggested as an optimal solution to facilitate the tapering discussions. On the other hand, patients expressed their concerns that they were excluded from the decision-making process about pain management during their clinic visits. Moreover, patients expressed their frustration with having passive clinical encounters and lack of providers’ interest in patients’ progress. These encounters had a negative impact on their commitment to therapy and their intention to return for follow-up visits with providers.

d. Interventions

Alternative therapy issues

Providers had positive views about physical therapy and described it as a good alternative to opioid therapy. However, they recognize that it requires frequent travel to the clinic and getting preauthorization approval from insurance. In addition to that, providers emphasized that the success of alternative therapy was highly contingent on patients’ willingness to accept the idea of tapering and trying non-opioid therapies. On the other hand, physical therapy was perceived negatively by patients. Several patients described inadequate pain control from approaches suggested at physical therapy sessions.

With respect to non-opioid medications, few providers suggested that non-opioid medications might be effective for reducing pain, but they had concerns about limited providers’ experience with emerging pharmaceuticals such as cannabidiol supplements and high risk of side effects of these medications. These reports were echoed by patients who expressed their concerns of the addictive properties of non-opioid medications such as gabapentin, and the difficulty of weaning off this type of medication.

Medication for opioid use disorder

As none of the participating providers were licensed to prescribe MOUD, issues related to MOUD were rarely discussed. Surgeons only reported the lack of guidance on managing patients undergoing surgery and taking methadone.

In contrast, discussions about challenges of obtaining MOUD and having individualized MOUD regimens were prominent during PT focus groups. Medications for opioid use disorder, such as Suboxone, were considered by patients to be an effective option to treat OUD. However, patients discussed concerns about being on Suboxone for too long and the rapid tapering process for Suboxone by some providers. Finding a provider trained and authorized to prescribe MOUD in the rural county was difficult for patients, as providers were mostly located in urban communities. The few providers in the county that were licensed to provide MOUD were overwhelmed with high patient caseloads and unable to meet the current needs.

#### 3.2.2. Mesolevel: Resources and Facilities

- Process of Care

Communication among staff/Coordination of care

There was a debate among providers about whose responsibility it is to address challenges related to high opioid doses and identifying potential opioid misuse. Inheriting patients who are taking high doses of opioids prescribed by other providers was cited as a challenge that many providers had to deal with when they first started their practice at their current clinic/hospital. Besides, primary care providers described seeing patients recovering from surgeries and seeking pain medications when they could not get refills for opioid prescriptions from their surgeons.

Disconnect between primary care teams and pain management teams was also reported. Inadequate documentation of the rational of maintaining patients on high opioid doses on the part of primary care physicians and referring patients without assessing their readiness to taper were reported by advanced pain management. These factors created tension and misunderstanding between patients and providers in advanced pain management. In contrast, few patients mentioned concerns about communication among care team. Those who did focused on the impact of poor coordination between emergency physicians and specialists on patients’ credibility and their ability to refill opioid prescriptions.

Timely access

Patients described the challenge of getting timely care from providers when facing unprecedent challenges. Not being able to connect with providers when needed resulted in patient self-initiated changes in prescription opioid use that might not be clinically appropriate and jeopardized patients’ ability to obtain opioid medication in the future.

b. Infrastructure

Behavioral Health and recovery resources

Both patients and providers acknowledged the importance of providing behavioral health services for OUD and chronic pain patients. Lack of mental health resources and shortage of psychiatrists and counselors in the country were considered significant barriers to holistic management of patients with OUD and chronic pain. Patients considered any clinical management (i.e., medication only) for OUD is inadequate and likely to fail. Underlying mental health problems such as depression and anxiety contribute to self-medicating and opioid abuse, thus they need to be supported by behavioral health interventions.

With regard to recovery centers, both patients and providers agreed that there is a limited number of nearby opioid treatment centers and patients were referred to centers outside the county to receive care. Patients described this as challenging for patients who were experiencing serious opioid withdrawal symptoms and having no options for immediate care that they could access to avoid relapse. Several patients expressed their need to “talk to someone”, especially when they were experiencing withdrawal symptoms in the past. Patients repeatedly suggested including, in the health care team, a recovery coach, who could likely understand their struggle and opioid use experience.

Information technology

Providers’ comments about technology limitations in the management of the opioid pandemic revolved around poor interoperability of different systems. For example, providers reported that the inability to integrate the Prescription Drug Monitoring Program (PDMP) with the electronic health record (EHR) created an extra burden for providers and reduced its adoption. Additionally, providers alluded to the limited ability of the PDMP to identify all patients with high morphine milligram equivalent (MME) who need follow-up and tapering.

On the other hand, patient framed information technology as a major contributor to stigma and discrimination by providers. From their perspective, having a permanent record of their OUD history in their electronic health records facilitated stigmatizing behavior by providers and blinded providers to the reality of the recovery status of patients.

#### 3.2.3. Macrolevel: Structural, Financial, and Legal Conditions of Care Provision

- Financing and reimbursement

Both patients and providers reported that getting insurance reimbursement for alternative therapies such as physical therapy and surgery was a common problem. Patients shared their frustration with the insurance policies that govern their pain management plan and affect their ability to wean opioid medications by limiting non-opioid options that are available for them. Similarly, physical therapists described that insurance companies cover a limited number of therapy sessions, which reduces the benefits that can be gained from these physical therapy treatments. Additionally, patients were required to pay high copays, which deters them from engaging in non-opioid treatments.

b. Policies, laws, and regulations

Several providers raised their concerns about organizational policies that evaluate physicians’ performance based on patient satisfaction surveys. Providers who declined to prescribe opioid medications were given poor ratings by patients, which reflected negatively on their performance evaluation. As a result, providers were more likely to engage in irrational opioid prescribing to avoid getting poor ratings in the future. Additionally, the absence of adequate oversight from the Food and Drug Administration (FDA) contributed to inappropriate opioid prescribing practices. However, providers expressed that recent changes in federal regulations on opioid prescribing have limited the opioid amount that providers can prescribe to patients nowadays, which can decrease the risk of misuse among patients. In addition to that, prescribers described their frustration with policies set by certain pharmacy chains that aim to limit patients supply of opioid medications despite their need. This was compounded by lack of proper communication between pharmacists and providers, which resulted in depriving patients from their opioid medication supply when they were in desperate need.

Patients had a general awareness that providers were affected by regulatory policies and guidelines, but seemed to be unaware of the details and largely did not discuss opioid policies and regulations.

Quotes about challenges to the management of chronic pain and treating OUD from both stakeholders are described in Table 1.

## 4. Discussion

The findings suggest that patients and providers in rural communities do not “read from the same script” most of the time regarding challenges to managing chronic pain and OUD. Although both patients and providers agreed that the lack of resources and insurance reimbursement for non-opioid interventions constitutes a major problem for managing pain and reducing opioid misuse, they had discordant opinions about other elements that contribute to inadequate chronic pain management and treatment for OUD.

### 4.1. Microlevel: The Interaction between Patient and Providers

Challenges described by patients in this study were focused mainly on the microlevel of care and the dynamics of patient–provider interaction. Providers’ discriminating behavior, inadequate knowledge and poor communication skills were the most highlighted barriers to achieving holistic, individualized patient centered care during PT focus groups, which resonates with other studies in the literature [27,28,29]. Patients in this study reported higher perceptions of stigma when receiving care in rural healthcare settings compared to urban settings. This is concerning, since providers’ stigma has been associated with lower providers’ likelihood to prescribe evidence-based treatments for OUD, lower support for policies that increase patients access to MOUD [30,31] and dismissing patients with chronic pain [32].

Labelling patients and documentation of their OUD history in a negative manner in the EHR seemed to play a role in promoting stigma. Stigmatizing language in medical records can propagate bias from one provider to another and was correlated with negative attitude towards patients and less aggressive treatment for patients’ pain [33]. Providers reported inadequate professional training and inability to distinguish between patients experiencing real pain from patients who were “drug seekers”, which might contribute to stigma among healthcare providers. This is unsurprising since federal laws and medical entities do not require training and competency in OUD evaluation and treatment in medical education [34]. Studies have shown that efforts to reduce stigma through continuing medical education contributed to less stigmatizing behavior among physicians [35]. Additionally, including recovery coaches in the management team can help reduce stigma and improve patients’ retention in care. Having gone through the same struggle and experienced recovery themselves, peer recovery coaches are uniquely positioned to support patients with OUD and reduce their social isolation [36].

Aside from stigma, patients expressed their frustration with the lack of shared decision making in treatment plans and providers’ failure to account for their preferences and needs. Studies have shown that patients who receive treatments that match their preferences had higher adherence rates and improvements in health outcomes [37]. In contrast, providers in this study reported difficulty in engaging patients in tapering decisions and described aggressive patients’ reaction to tapering requests. This contradiction between patients and providers could be attributed to two factors: characteristics of patients in this study and the communication style of providers when discussing tapering with patients. Most of the patients recruited to this study were informed and interested in playing an active part in their health care. However, they constitute a small subset of patients that providers see in their practice. On the other hand, during interviews with different providers, drastic differences were noted in providers’ approach and the way they initiate tapering conversations with patients. For example, some providers recommended tapering for all patients who had daily MME above 90, whereas other providers described the importance of building a relationship with patients and assessing their functional status and pain control before initiating tapering discussions. Evidence from the literature suggests that a unilateral approach to tapering by physicians often leads to anger and hostility by patients [38]. Even worse, opioid tapering can lead to termination of care [39]. Discussions around tapering should be centered around individuals and their clinical circumstances and focused on the positive benefits of abstinence rather than on the negative impact of addiction. Resistance to tapering needs to be met with empathy, negotiation of treatment plans and goals, ongoing conversations and showing support and non-abandonment [40]. This approach was reflected in the few successful tapering stories that were shared by providers in the focus group, which resulted from providing patient-centered care such as building partnership with patients, providing more information, and individualization of tapering.

### 4.2. Meso- and Macrolevel of Care

Although challenges related to getting access to MOUD and the lack of physician training in providing MOUD were prominent themes during PT discussions, these concerns were not reciprocated by providers in the focus group sessions. This discordance in priorities among patients and providers can be attributed to the fact that none of the participating physicians had training or experience with prescribing MOUD. Competing demands, lack of leadership support, staff stigma, and physicians’ disinterest in getting licensed to avoid scrutinization by Drug Enforcement Administration were reported in the literature as barriers to getting federal Drug Addiction Treatment Act of 2000 (DATA) waiver [41,42,43]. Providers’ attitudes towards MOUD in this study is unclear and needs to be assessed in future studies. Evidence from published literature show that MOUD therapies such as buprenorphine and naltrexone are effective in reducing cravings, withdrawal symptoms, and relapse in patients with OUD [44,45]. Challenges to providing MOUD in rural communities need to be addressed by providing support and training to providers and encouraging other health care professionals such as nurse practitioners and physician assistants to obtain waiver privileges.

Disproportionate interest in alternative therapies for managing chronic pain was also evident during discussions with patients and providers. Challenges to access alternative therapies reported by patients such as high out-of-pocket cost and long-distance travel to clinics can explain to some extent patients’ reluctance to try non-opioid therapies suggested by providers. Poor use of alternative therapies is further compounded by a lack of coordination between different providers and delivery of mixed messages to patients about treatment goals. These challenges are considered substantial and require multidimensional strategies to enhance collaboration between providers and reduce system barriers to access non-opioid alternatives.

The imbalance in opinions about the influence of policies and regulations on the management of chronic pain and/or OUD is inherent in patients’ and providers’ roles. Providers are under significant pressure to achieve high patients’ satisfaction ratings, limit the loss of patients, and to refer patients with OUD to treatment despite institutional policies that facilitate such referral. Each of these factors shape the treatment decisions made by providers. Providers are then viewed as unprofessional and unempathetic by patients who likely are not aware of the myriad of forces that are influencing provider decision-making “behind the scenes”.

In conclusion, high discrepancy was noted among patients and providers regarding barriers and facilitators to the management of chronic pain and treatment for OUD. Including the perspectives of both stakeholders is critical when developing strategies to confront the opioid crisis in rural communities. Increasing the number of behavioral health resources without providing adequate access to MOUD will produce minimal progress in reducing the toll of opioid misuse and overdose. Similarly, expanding the number of DATA waived providers and providing more training and education in tapering is crucial, but none more important than efforts to reduce stigma embedded in the rural healthcare communities. Addressing the opioid crisis in rural areas is challenging and requires a diverse multifaceted approach. The Project ECHO model that was implemented in rural New Mexico is considered a modified “hub and spoke” approach that is best suited for rural areas where treatment services and opioid treatment program hubs are limited. In this model, primary providers receive specialized training and support in opioid prescribing and MOUD management through telehealth services [46]. The strength of this model is the emphasis on psychosocial services and team-based approach to facilitate patients’ screening and monitoring. Including other healthcare professionals such as pharmacists and physician assistants in chronic pain and OUD management team can increase patients’ access to health services and reduce workload on prescribers. This model can be further leveraged to meet patients’ needs for social support and connectedness by including recovery coaches in the management team. Implementing team-based models for the treatment of OUD while accounting for the unique characteristics of rural settings has the potential to expand patients’ access to effective treatments and overcome many of the challenges that were described by stakeholders in this study.

### 4.3. Strengths and Limitations

This study has several limitations. First, the number of participating patients was relatively small and represented a subset of patients who have recovered from OUD and/or who are living with chronic pain, which prevents the generalizability of the findings to other patient populations who have ongoing OUD. Second, both participant groups lacked racial and ethnic diversity, which precludes the experiences and perceptions of patients and providers from other ethnic backgrounds. However, participants’ demographics reflect the current population in rural Wisconsin that is dominated by Whites [47]. There was also limited representation of providers who primarily manage chronic pain. As this is a secondary analysis of primary qualitative data, key information might have been missed as we could not conduct further interviews to get more clarification or validate the findings by member checking. Additionally, it was not possible to determine whether thematic saturation was achieved with the small number of focus groups and interviews that were conducted. However, findings of this study are consistent with previous literature on the experiences of patients with OUD and chronic pain which increases our confidence in the findings. Moreover, counts and frequencies of the qualitative codes were not reported in the findings as it’s laborious and time-consuming process with manual-coding. Strengths of the study include good participant retention rates across FP and PT focus groups, and low power differential among participants in the PT group, which allowed participants to fully express their opinions and perceptions. Furthermore, participants in the FP group were involved in an opioid taskforce at FHC and have genuine interest in identifying gaps and opportunities to manage chronic pain and OUD.

## 5. Conclusions

In resource-scarce communities, providing patient-centered care is challenging and requires an interdisciplinary approach. Including perspectives of both patients and providers is critical when developing strategies to confront the opioid crisis. Although most of past research has focused on expanding patients’ access to effective treatments and establishing more recovery facilities for patients with OUD, there should be equal efforts to mitigate stigma among providers in rural communities, support psychosocial needs of patients, and build trusting relationships with patients.

## Figures and Tables

**Table 1:** Quotes from study participants about challenges to the management of chronic pain and OUD in rural Wisconsin.

Aspects of Each Level of Care	Group	Quotes
***Providers’ knowledge, attitude, and professional skills***	**Patients**	“And I remember saying to the doctor, Dr. [NAME], he’s going to get addicted, because he had him on like five different pain meds. And Dr. [NAME] said, on, don’t worry, this, in a little, we will get him off.” *Patient 6* “The man literally took two steps back from me, like I had leprosy. And the first sentence out of his mouth was, I am not giving you any pills.” *Patient 3* “They can believe a broken arm, a broken leg, wisdom teeth, whatever. They can believe in that. But chronic pain is something new to a lot of people.” *Patient 2* “And to their credit, addicts are, we’re liars, we’re cheats, we’re thieves.” *Patient 3*
	**Providers**	“I’m going back to your chart and so I went back like five years before I found out that she hurt her back weeding her garden, and someone started her out on Tylenol-Codeine then they just kept increasing the dose.” *Physician assistant* *Written on sticky note:* How to handle patient who never seems able to get drug screen done or pill count done; get mixed message about discharge of patient (for failure of contract) vs. we do not want to lose patient from clinic
***Patients’ knowledge, attitude, and personal characteristics***	**Patients**	“And I don’t want to be on opioids. But I don’t want to feel the results of being off of it either.” *Patient 4* “We all have assumptions of whatever. But we need to be looked at as human beings, mind, body, and soul.” *Patient 3*
	**Providers**	“Yeah, well, “why can’t I have more ?” and then explain why we can’t have more and then they seem to understand that but they also don’t seem to understand that if you have too much, that can make the pain worse and if we back down, it might be worse for a couple of weeks but then it might actually be better it’s like they’re too afraid to try that.” *Pharmacist 1* “What I’ve been finding it’s like how do I break through these roadblocks to get people to see that maybe there is a benefit” *Pharmacist 1*
***Patient-provider communication***	**Patients**	“Doctors kind of, do it this way, and this is the way they say it should be done, when it really should and that’s where people tend to drop off and fall off here and there in the system, because that particular way is just not working for them.” *Patient 5*
	**Providers**	“ I think maybe our doctors aren’t really having that conversation with the patient. They keep saying, I would like you to go down I’m not going to fill more for you.” *Pharmacist 1* “and she said, like the relationship she had with her previous providers, they just kind of come in, refill them and be like okay they’re working fine and then go.” *Pain management practitioner* “postoperative pain management is important. You know, new people come in, being put on narcotics. If we could, you know, there are people who are going to need them, without a doubt. But if you set expectations, we set the timeframe right, that we set everybody straight so that we prevent those people from progressing into addiction.” *Hospitalist*
***Medication for opioid use disorder***	**Patients**	“I think Suboxone can be a great treatment, but it’s not, they’re not using it as, I believe, that you’re supposed to. You know, it’s supposed to be a gateway to get off them, but they become long term. My son has been on it for seven years.” *Patient 4* “and he went in to the hospital to get off the Suboxone because he didn’t want to be in that spot any longer. It was too easy to abuse. And that’s what he was doing. So I actually just talked to him today, and he’s doing really good. He’s off it completely. But we’ve talked a lot about how coming off of Suboxone was harder for him than coming off heroin.” *Patient 6* “And something I wrote down is I personally believe that Suboxone should be more available. Just speaking of me trying to get off heroin starting in like 2012, I think. It was so hard to find a provider.” *Patient 3*
	**Providers**	*Written on sticky note:* “Surgical patients on methadone therapy: how to manage pre-surgery, intra surgery and post survey, how to warn anesthesia team.”
***Communication among staff and Coordination of care***	**Patients**	“The ER never called pain management. I almost lost my prescription because they had given me more than what I was allowed to try to get me back down to the pain level.” *Patient 2*
	**Providers**	“so we just told the patient, you know, we don’t know but we see that you’ve been getting scripts from your primary so like we are not touching that because we don’t want to step on anybody’s toes. Turns out the primary did want us.” *Pain management nurse*
***Timely access***	**Patients**	“So now I’m down to two pills a day instead of four. But that’s not working for me. Can I try three? But it might take the provider a week to get back to me, right?” *Patient 1* “And I’ll tell you for myself, being an addict, you give me more than 12 h, I’m going back… so the fact that, you know, you give them some hope, but then, well, they have a bed, but it won’t be available until such and such.” *Patient 4*
	**Providers**	NA
***Alternative therapy issues***	**Patients**	“Physical therapy doesn’t do anything. It just, it makes it just flare up and that. So I have radiofrequency coming up in September because that’s, that’s not for my bulge. That’s for my arthritis that I have on four disks I’m having for that. But my bulge, I had a cortisone shot done. That didn’t do nothing for it.” *Patient 2*
	**Providers**	“We have really good physical therapy department. Yeah, they do more than just exercises. They have all kinds of modalities they, they’re very, very good. Again, if people would go in there open minded. Most of them actually get help.” *Physician assistant*
***Behavioral health and recovery resources***	**Patients**	“And my other issue with [……] County in general, so gigantic, a lack of mental healthcare services. I currently see a psychiatrist and therapist at [……] County Human Services. They are swamped. We need more facilities.” *Patient 3*
	**Providers**	“I think, as an organization, we need to do a better job of recruiting for behavioral health professionals. And I get that it’s hard. It’s a hot area right now.” *Pharmacist 1*
***Information technology***	**Patients**	“It’s on your record, and it doesn’t matter if you’ve been clean for ten years. It shows up on your record, and pain management is all nice, nice, nice, until they scroll further down and read your, and see that it happened in the past, ten years ago. And says, oh, I’m not giving you any pills.” *Patient 3*
	**Providers**	“I’ll just add on the PDMP, I think this is an immensely powerful tool that is underutilized. And part of that is not due to anybody’s fault. It’s due to our inability to integrate it with the EHR. So you got this immensely powerful tool that is clunky to use.” *Pharmacist 2*
***Financing and reimbursement***	**Patients**	“The insurance companies, they make you go through loophole after loophole after loophole… you got to go through ten different things before the insurance company finally says, hey, why don’t we give that, do that procedure for that person to begin with? *Patient 2*
	**Providers**	“like we build them up and say, oh, drugs aren’t everything, drugs aren’t everything. And then they’re, we say, oh, we changed our minds. Since it won’t be covered by insurance, drugs are everything.” *Pharmacist 1*
***Policies, laws, and regulations***	**Patients**	NA
	**Providers**	“[Pharmacy name] told me if an MME is over 50, they will not dispense it, period.” *Pharmacist 1* “Can I add one thing on the expectation that I think works against us sometimes is we are measured on patient satisfaction based on pain control. And I think, in a lot of aspects, that works against us when you’re trying to set good expectations. There’s a question on the patient survey, did we control their pain, or something to that effect.” *Pharmacist 2* “I think that if the FDA start sending out letters like they have been. That might be a wakeup call for, some providers, right, because they will be like Oh my god! they’re watching! Because they are like, outside the realm of what’s considered good practice.” *Physician assistant*

MME: morphine milligram equivalent, PDMP: Prescription Drug Monitoring Program.

## Data Availability

Data can be provided by the corresponding author upon request.

## References

[1] Chronic Pain Management and Opioid Misuse: A Public Health Concern (Position Paper). https://www.aafp.org/about/policies/all/chronic-pain-management-opiod-misuse.html.

[2] Ahmad F.B., Rossen L.M., Sutton P. (2021). Provisional Drug Overdose Death Counts.

[3] Luo F., Li M., Florence C. (2021). State-Level Economic Costs of Opioid Use Disorder and Fatal Opioid Overdose—United States, 2017. Morb. Mortal. Wkly. Rep..

[4] Andrew R., Derry S., Taylor R.S., Straube S., Phillips C.J. (2014). The costs and consequences of adequately managed chronic non-cancer pain and chronic neuropathic pain. Pain Pract. Off. J. World Inst. Pain.

[5] Moriarty O., McGuire B.E., Finn D.P. (2011). The effect of pain on cognitive function: A review of clinical and preclinical research. Prog. Neurobiol..

[6] Mack K.A., Jones C.M., Ballesteros M.F. (2017). Illicit Drug Use, Illicit Drug Use Disorders, and Drug Overdose Deaths in Metropolitan and Nonmetropolitan Areas—United States. Am. J. Transplant..

[7] Wisconsin Department of Health Services Preventing and Treating Harms of the Opioid Crisis: An Assessment to Identify Geographic Gaps in Services and a Plan to Address These Gaps. https://www.dhs.wisconsin.gov/publications/p02605.pdf.

[8] Rural Voices for Prosperity: A Report of the Governor’s Blue Ribbon Commission on Rural Prosperity. Web_Governors-Blue-Ribbon-Commission-Report.pdf.

[9] Keyes K.M., Cerdá M., Brady J.E., Havens J.R., Galea S. (2014). Understanding the Rural-Urban Differences in Nonmedical Prescription Opioid Use and Abuse in the United States. Am. J. Public Health.

[10] Franz B., Dhanani L.Y., Miller W.C. (2021). Rural-Urban Differences in Physician Bias toward Patients with Opioid Use Disorder. Psychiatr. Serv..

[11] van Boekel L.C., Brouwers E.P., van Weeghel J., Garretsen H.F. (2013). Stigma among health professionals towards patients with substance use disorders and its consequences for healthcare delivery: Systematic review. Drug Alcohol Depend..

[12] Slade S.C., Molloy E., Keating J.L. (2009). Stigma Experienced by People with Nonspecific Chronic Low Back Pain. Pain Med..

[13] De Ruddere L., Goubert L., Stevens M., de Williams A.C.C., Crombez G. (2013). Discounting pain in the absence of medical evidence is explained by negative evaluation of the patient. Pain.

[14] Bergman A.A., Matthias M.S., Coffing J.M., Krebs E.E. (2013). Contrasting tensions between patients and PCPs in chronic pain management: A qualitative study. Pain Med..

[15] Joosten E.A., De Weert-Van Oene G.H., Sensky T., Van Der Staak C.P., De Jong C.A. (2011). Treatment goals in addiction healthcare: The perspectives of patients and clinicians. Int. J. Soc. Psychiatry.

[16] Alves P., Sales C., Ashworth M. (2017). Does outcome measurement of treatment for substance use disorder reflect the personal concerns of patients? A scoping review of measures recommended in Europe. Drug Alcohol Depend..

[17] Stewart M., Brown J.B., Donner A., McWhinney I.R., Oates J., Weston W.W., Jordan J. (2000). The impact of patient-centered care on outcomes. J. Fam. Pract..

[18] Fiscella K., Meldrum S., Franks P., Shields C.G., Duberstein P., McDaniel S.H., Epstein R.M.J.M.C. (2004). Patient trust: Is it related to patient-centered behavior of primary care physicians?. Med. Care.

[19] Spinuzzi C. (2005). The Methodology of Participatory Design. Tech. Commun..

[20] Macaulay A.C., Commanda L.E., Freeman W.L., Gibson N., McCabe M.L., Robbins C.M., Twohig P.L. (1999). Participatory research maximises community and lay involvement. BMJ.

[21] Chui M.A., Stone J.A., Holden R.J. (2017). Improving over-the-counter medication safety for older adults: A study protocol for a demonstration and dissemination study. Res. Soc. Adm. Pharm. RSAP.

[22] Wisconsin Network for Research Support. https://winrs.nursing.wisc.edu/.

[23] Strauss A., Corbin J. (1998). Basics of Qualitative Research: Techniques and Procedures for Developing Grounded Theory.

[24] Gale N.K., Heath G., Cameron E., Rashid S., Redwood S. (2013). Using the framework method for the analysis of qualitative data in multi-disciplinary health research. BMC Med. Res. Methodol..

[25] Vennedey V., Hower K.I., Hillen H., Ansmann L., Kuntz L., Stock S. (2020). Patients’ perspectives of facilitators and barriers to patient-centred care: Insights from qualitative patient interviews. BMJ Open.

[26] Boyatzis R. (1998). Transforming Qualitative Information: Thematic Analysis and Code Development.

[27] Goodyear K., Haass-Koffler C.L., Chavanne D. (2018). Opioid use and stigma: The role of gender, language and precipitating events. Drug Alcohol Depend..

[28] Woo J., Bhalerao A., Bawor M., Bhatt M., Dennis B., Mouravska N., Zielinski L., Samaan Z. (2017). “Don’t Judge a Book Its Cover”: A Qualitative Study of Methadone Patients’ Experiences of Stigma. Subst. Abus. Res. Treat..

[29] Ezell J.M., Walters S., Friedman S.R., Bolinski R., Jenkins W.D., Schneider J., Link B., Pho M.T. (2021). Stigmatize the use, not the user? Attitudes on opioid use, drug injection, treatment, and overdose prevention in rural communities. Soc. Sci. Med..

[30] Stone E.M., Kennedy-Hendricks A., Barry C.L., Bachhuber M.A., McGinty E.E. (2021). The role of stigma in U.S. primary care physicians’ treatment of opioid use disorder. Drug Alcohol Depend..

[31] Rao D., Giannetti V., Kamal K.M., Covvey J.R., Tomko J.R. (2021). The relationship between knowledge, attitudes, and practices of community pharmacists regarding persons with substance use disorders. Subst. Abus..

[32] Tools for Stakeholder Engagement in Research. https://www.hipxchange.org/StakeholderEngagementTools.

[33] Goddu A.P., O’Conor K.J., Lanzkron S., Saheed M.O., Saha S., Peek M.E., Haywood C., Beach M.C. (2018). Do Words Matter? Stigmatizing Language and the Transmission of Bias in the Medical Record. J. Gen. Intern. Med..

[34] Weimer M.B., Wakeman S.E., Saitz R. (2021). Removing One Barrier to Opioid Use Disorder Treatment: Is It Enough?. J. Am. Med. Assoc..

[35] Kennedy-Hendricks A., Barry C.L., Stone E., Bachhuber M.A., McGinty E.E. (2020). Comparing perspectives on medication treatment for opioid use disorder between national samples of primary care trainee physicians and attending physicians. Drug Alcohol Depend..

[36] Cabral R.R., Smith T.B. (2011). Racial/ethnic matching of clients and therapists in mental health services: A meta-analytic review of preferences, perceptions, and outcomes. J. Couns. Psychol..

[37] Swift J.K., Callahan J.L. (2009). The impact of client treatment preferences on outcome: A meta-analysis. J. Clin. Psychol..

[38] Frank J.W., Levy C., Matlock D.D., Calcaterra S.L., Mueller S.R., Koester S., Binswanger I.A. (2016). Patients’ Perspectives on Tapering of Chronic Opioid Therapy: A Qualitative Study. Pain Med..

[39] Matthias M.S. (2020). Opioid Tapering and the Patient-Provider Relationship. J. Gen. Intern. Med..

[40] Davis M.P., Digwood G., Mehta Z., McPherson M.L. (2020). Tapering opioids: A comprehensive qualitative review. Ann. Palliat. Med..

[41] Kissin W., McLeod C., Sonnefeld J., Stanton A. (2006). Experiences of a national sample of qualified addiction specialists who have and have not prescribed buprenorphine for opioid dependence. J. Addict. Dis..

[42] Hutchinson E., Catlin M., Andrilla C.H.A., Baldwin L.-M., Rosenblatt R.A. (2014). Barriers to Primary Care Physicians Prescribing Buprenorphine. Ann. Fam. Med..

[43] Madden E.F. (2019). Intervention stigma: How medication-assisted treatment marginalizes patients and providers. Soc. Sci. Med..

[44] Thomas C.P., Fullerton C.A., Kim M., Montejano L., Lyman D.R., Dougherty R.H., Daniels A.S., Ghose S.S., Delphin-Rittmon M.E. (2014). Medication-assisted treatment with buprenorphine: Assessing the evidence. Psychiatr. Serv..

[45] Jarvis B.P., Holtyn A.F., Subramaniam S., Tompkins D.A., Oga E.A., Bigelow G.E., Silverman K. (2018). Extended-release injectable naltrexone for opioid use disorder: A systematic review. Addiction.

[46] Komaromy M., Duhigg D., Metcalf A., Carlson C., Kalishman S., Hayes L., Burke T., Thornton K., Arora S. (2016). Project ECHO (Extension for Community Healthcare Outcomes): A new model for educating primary care providers about treatment of substance use disorders. Subst. Abus..

[47] QuickFacts: Wisconsin. https://www.census.gov/quickfacts/WI.

